# QTL mapping and transcriptome analysis identify candidate genes regulating pericarp thickness in sweet corn

**DOI:** 10.1186/s12870-020-2295-8

**Published:** 2020-03-14

**Authors:** Xiaming Wu, Bo Wang, Fugui Xie, Liping Zhang, Jie Gong, Wei Zhu, Xiaoqin Li, Faqiang Feng, Jun Huang

**Affiliations:** 1 The State Key Laboratory for Conservation and Utilization of Subtropical Agro-bioresources, South China Agricultural University, Guangzhou, 510642 Guangdong People’s Republic of China; 2 The Key Laboratory of Plant Molecular Breeding of Guangdong Province, College of Agriculture, South China Agricultural University, Guangzhou, 510642 Guangdong People’s Republic of China

**Keywords:** Sweet corn, Pericarp thickness, QTL, Transcriptome, SLAF tags

## Abstract

**Background:**

In recent years, the planting area of sweet corn in China has expanded rapidly. Some new varieties with high yields and good adaptabilities have emerged. However, the improvement of edible quality traits, especially through the development of varieties with thin pericarp thickness, has not been achieved to date. Pericarp thickness is a complex trait that is the key factor determining the edible quality of sweet corn. Genetic mapping combined with transcriptome analysis was used to identify candidate genes controlling pericarp thickness.

**Results:**

To identify novel quantitative trait loci (QTLs) for pericarp thickness, a sweet corn BC_4_F_3_ population of 148 lines was developed using the two sweet corn lines M03 (recurrent parent) and M08 (donor parent). Additionally, a high-density genetic linkage map containing 3876 specific length amplified fragment (SLAF) tags was constructed and used for mapping QTLs for pericarp thickness. Interestingly, 14 QTLs for pericarp thickness were detected, and one stable QTL (*qPT10–5)* was detected across multiple years, which explained 7.78–35.38% of the phenotypic variation located on chromosome 10 (144,631,242-145,532,401). Forty-two candidate genes were found within the target region of *qPT10–5*. Moreover, of these 42 genes, five genes (*GRMZM2G143402*, *GRMZM2G143389*, *GRMZM2G143352*, *GRMZM6G287947*, and *AC234202.1_FG004*) were differentially expressed between the two parents, as revealed by transcriptome analysis. According to the gene annotation information, three genes might be considered candidates for pericarp thickness. *GRMZM2G143352* and *GRMZM2G143402* have been annotated as AUX/IAA transcription factor and ZIM transcription factor, respectively, while *GRMZM2G143389* has been annotated as FATTY ACID EXPORT 2, chloroplastic.

**Conclusions:**

This study identified a major QTL and candidate genes that could accelerate breeding for the thin pericarp thickness variety of sweet corn, and these results established the basis for map-based cloning and further functional research.

## Background

Sweet corn is a maize-derived vegetable crop developed through one or several recessive endosperm mutations that reduce the synthesis of starch and increase the accumulation of sugars or other short-chain polysaccharides. The growing area of sweet corn has increased rapidly in China. In recent years, some new varieties with high yields and good adaptabilities have emerged. However, improvement of edible quality traits, especially through the development of varieties with reduced pericarp thickness, has not been achieved to date. Kernel tenderness, crispness and residue percentage of sweet corn are important criteria for evaluating edible quality. Pericarp thickness and structure are closely related to these three quality traits. Thin pericarps show high softness, high crispness, low residue rate, and good taste [1]. The pericarp of corn is composed of nondigestible cellulose, which is neither nutritious nor digestible, and the thickness of the pericarp affects the tenderness of sweet corn. Thus, the selection of a thinner pericarp is highly important in sweet corn quality breeding programs. Reducing pericarp thickness has become an important breeding goal to improve the edible quality of sweet corn.

The genetic characteristics of maize pericarp thickness have a high narrow sense heritability (55–82%) and involve additive effects, dominant effects and significant epistasis effects [2–4]. The number of effective genes associated with pericarp thickness estimates averaged 3.3 and ranged from 1.4 to 5.9 for the eight crosses [4]. To date, no single gene with a major effect (> 25%) has been identified. Haddad showed that the cell layer number of maize hybrids was the same as that of their female parents, and the difference in pericarp thickness was due to the thickening of the cell wall of the hybrids, which indicated that the thickness of the maize pericarp was affected by both maternal and nuclear genes [5]. Tracy and Schmidt analysed the pericarp thickness of 7 different sweet corn near-isogenic lines that differed in endosperm composition [*sugary* (*su)*, *dull* (*du*), *waxy* (*wx*), *sugary-2* (*su2*), and *shrunken-2* (*sh2*)] using a pressure micrometer and found that the pericarp thickness was significantly affected by embryo type, endosperm type, endosperm by inbred interactions, ear, and position of measurement on the kernel [6]. A study by Helm and Zuber showed that the narrow heritability of pericarp thickness was 0.80 and that pericarp thickness was controlled by 3 to 8 semidominant, epistatic and additive genes [2]. ITO and Brewbaker showed that pericarp thickness was controlled by 2–5 semidominant genes [4]. Therefore, the gene loci and their genetic effects controlling pericarp thickness may be more easily identified and analysed by molecular methods.

To the best of our knowledge, few studies on linkage mapping for maize pericarp thickness have been reported. For example, eight chromosomal fragments affecting sweet corn pericarp thickness were identified using restriction fragment length polymorphism (RFLP) markers in a chromosome segment substitute lines (CSSL) population [7]. Wang and Brewbaker used 94 maize recombinant inbred lines and identified three pericarp thickness QTLs located on chromosomes 1, 2 and 6 [8]. Forty-one QTLs related to pericarp thickness were detected using 100 simple sequence repeat (SSR) markers. Moreover, principal component analysis indicated that the first principal component composed of 8 QTLs could explain 87.60% of pericarp thickness phenotypic variance, which could be applied in breeding thin pericarp maize varieties [9, 10]. Choe and Rocheford TR also found that some QTLs controlling pericarp thickness in a waxy corn mapping panel were colocated with ear trait QTLs, which may be due to the high correlation between pericarp thickness and ear traits [11]. Eight pericarp thickness QTLs were detected based on two genetic models using the 190 BC_1_F_2_ population. Three QTLs for pericarp thickness were identified using the composite interval mapping (CIM) method and explained 8.6, 16.0, and 7.2% of phenotypic variation, respectively. Based on the mixed model-based CIM (MCIM) method, five QTLs for pericarp thickness were detected [12]. Although these investigations have been undertaken, no pericarp thickness genes have been cloned to date. These genetic maps commonly have low marker density, which makes it difficult to cover the whole genome, thereby making QTL analysis difficult. Next-generation sequencing (NGS) technology makes it possible to detect large quantities of SNP markers covering the entire genome. Specific length amplified fragment sequencing (SLAF-seq), first developed by Sun et al. [13], has been widely used for high-density genetic map construction, such as cotton [14], grape [15], and cucumber [16]. Compared with traditional molecular markers, the distribution density of markers in genetic mapping affects the accuracy of the mapping, and the higher the density is, the more accurate the mapping is. In addition, SLAF-seq has overcome the shortcomings of traditional markers, which are more time-consuming and labour-intensive. Therefore, SLAF-seq has been considered as a cost-effective technique to develop high stability and specificity molecular markers.

At present, tasting is a prevalent method for the comprehensive evaluation of sweet corn varieties by breeders. Although this method is direct and practical, its disadvantages include strong subjectivity and inaccurate phenotyping, making gene cloning difficult. Currently, with the rapid development of multiomics technologies (e.g., genomics, transcriptomics, metabolomics, proteomics, epigenomics, and lonomics), there are new opportunities to explore genes involved in the formation of pericarp thickness. Transcriptomics has been shown to be a powerful tool for the large-scale identification of genes related to specific traits in some crop species, including rice [17], maize [18], wheat [19], barley [20], and cotton [21]. To date, no RNA-seq study of pericarp thickness has been reported; thus, a more effective method to identify genes related to pericarp thickness is developed by integrating linkage analysis and transcriptome analysis. To identify the genes controlling the pericarp thickness of sweet corn and understand the genetic basis of the development of pericarp thickness in sweet corn. In this study, we constructed a BC_4_F_3_ population using two sweet corn inbred lines with different pericarp thicknesses. Therefore, our aims are 1) to map pericarp thickness QTLs by linkage mapping and 2) to propose candidate genes for those QTLs based on complementary transcriptomic analyses. These results may provide molecular markers for sweet corn breeding with thinner pericarps and a theoretical basis for quality improvement and industrialization of sweet corn.

## Results

### Phenotypic analysis

The phenotypic data of the pericarp thickness of the 148 BC_4_F_3_ population and their two parents were collected in autumn of 2014, 2015 and 2016. As shown in Table 1, the pericarp thickness showed significant differences between the two parental lines across the 3 years. Compared with the ‘M08’ inbred line, the paternal parent ‘M03’ inbred line exhibited a lower pericarp thickness. The pericarp thickness of the BC_4_F_3_ population ranged from 30.63 to 104.21 μm and displayed a continuous distribution. The skewness and kurtosis of the trait ranged from 0.78–1.23 and 1.17–2.29, respectively, and the broad sense heritability (*h*^2^) of 3 years and cross years ranged from 0.66 to 0.73 (Table 1). The distributions of pericarp thickness for the BC_4_F_3_ population were determined for samples over the 3 years and suggested that the segregation of this trait approximates the normal distribution and indicated that the pericarp thickness is a typical quantitative trait that is controlled by polygenes. A continuous distribution and considerable transgressive segregation were shown in the BC_4_F_3_ population, suggesting that both parents contributed alleles towards pericarp thickness.

**Table 1:** Descriptive statistics of pericarp thickness for the parental lines and BC_4_F_3_ populations

Trait	Parents (Mean ± SD)		BC_4_F_3_ Population			Skewness	Kurtosis	*h*^2^
	M03^a^	M08^a^	Minimum^a^	Maximum^a^	Mean ± SD^a^			
2014 FS	57.86 ± 7.28	95.07 ± 6.36	46.17	104.21	70.80 ± 10.17	0.78	1.17	0.68
2015 FS	52.48 ± 10.22	97.81 ± 13.36	45.28	104.20	66.68 ± 10.57	0.91	2.29	0.66
2016 FS	47.29 ± 11.23	80.55 ± 13.28	30.63	94.66	50.04 ± 12.31	1.23	2.09	0.73
Average years	52.54 ± 9.58	91.14 ± 11.01	44.19	99.26	67.41 ± 10.81	1.09	1.47	0.67

^a^ μm

*h*^2^ refer to broad sense heritability

A linear mixed model with random genotype three year interaction was used for analysis across three years

Positive correlations were observed among the traits evaluated over the 3 years (Additional file 1: Table S1). Correlation coefficients of pericarp thickness from the 3 years were found to be significant (*P* < 0.01) and were correlated with each other in a range from *r* = 0.756 to 0.915. This result indicates that the pericarp thickness was stable among different environments.

### Identification of major pericarp thickness QTLs in the BC_4_F_3_ population

DNA of the 148 BC_4_F_3_ population was sent to a biomarker company for SLAF sequencing. A total of 163,961 SLAF tags were obtained, in which the coverage depth of the two parents was 42.15**×**, and the average sequencing depth of the offspring was 5.47×. To improve the quality of the genetic map, SLAFs were filtered according to Zhu’s method: 1) parents sequence depth < 10×; 2) complete degree < 30%; 3) partial separation significantly (*P* < 0.01); 4) heterozygous in two parents [16].

A total of 3876 SNPs were obtained after filtering the original SLAF tags. Based on the locations of SLAF markers on chromosomes, they were assigned into ten linkage groups (LGs) according to the maize inbred line B73 reference genome (version 3) (ftp://ftp.ensemblgenomes.org/pub/plants/release-24/fasta/zea_mays/dna/). The linear arrangement of markers in the linkage group was obtained using JoinMap4.1 software, and the genetic distance between adjacent markers was estimated. Finally, a genetic map with a total map distance of 2413.25 cM was constructed. The number of markers on the map was 3876, and the average map distance between markers was 0.62 cM (Additional file 2: Fig. S1; Additional file 3: Table S2).

**Fig. 1:**
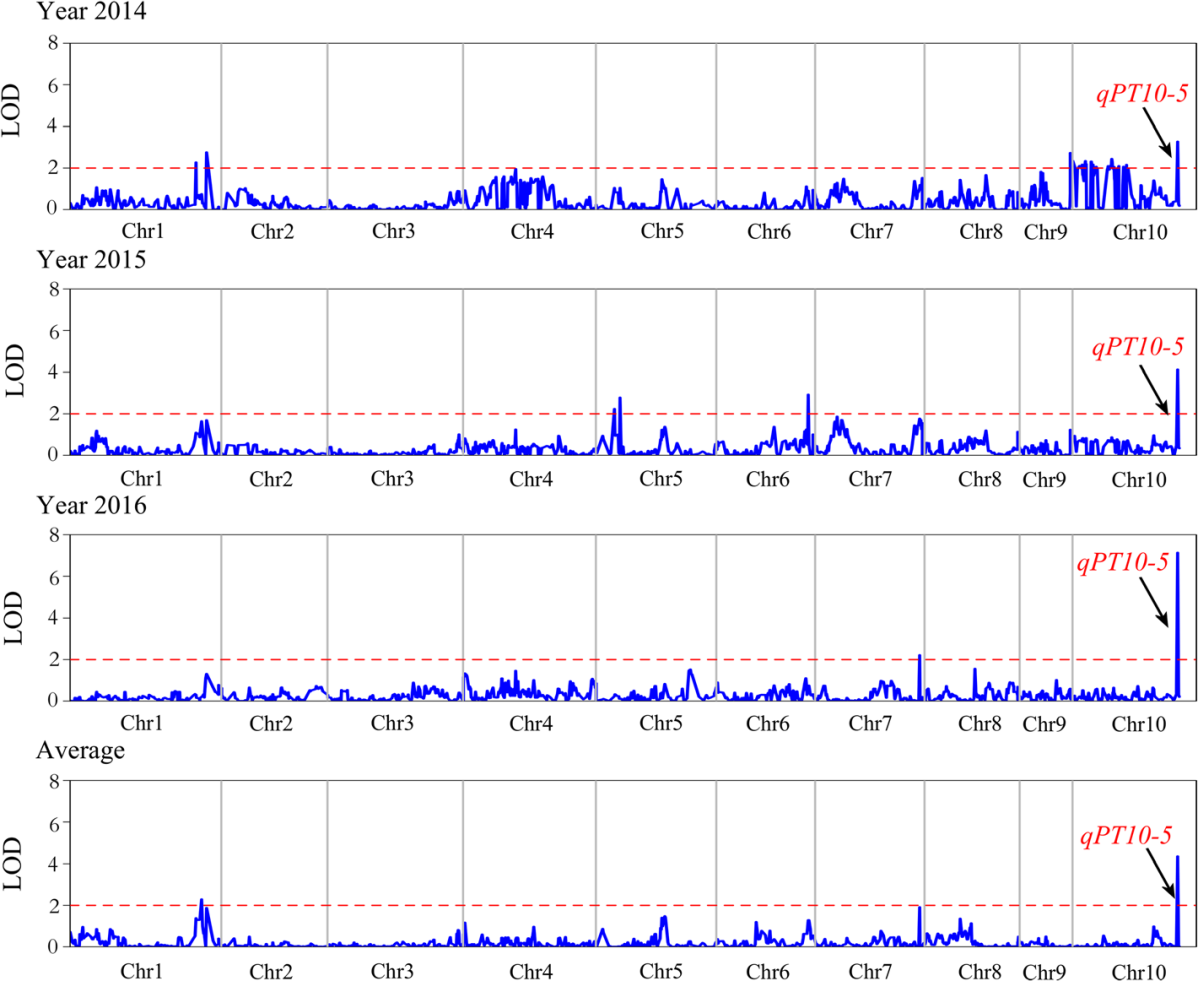
Single-trait multiple-interval mapping of the QTL for pericarp thickness on ten chromosomes across three years in the BC_4_F_3_ population. Curves in the plot indicate the genetic coordinates along chromosomes within a chromosome (x-axis) and LOD score (y-axis) of the detected QTL. The mapping curve of the QTL that controls pericarp thickness is located on chromosome 10, and a common *qPT10–5* was detected across three years. The dashed line indicates the significance threshold (LOD = 2.0)

Based on the constructed genetic linkage map, the phenotype of the pericarp thickness of the BC_4_F_3_ population collected from 2014, 2015, 2016 and the average of those 3 years were analysed by QTL mapping. A total of 14 QTLs for pericarp thickness were mapped in the BC_4_F_3_ populations across 3 years. The QTLs were distributed on maize chromosomes 1, 5, 6, 7, and 10. Seven QTLs associated with pericarp thickness were found on chromosomes 1 and 10, and the phenotypic variance explained by a single QTL ranged from 3.36 to 7.78% in 2014. Three QTLs controlling pericarp thickness were identified on chromosomes 5, 6, and 10, accounting for 26.32% of phenotypic variance in 2015. Two QTLs (*qPT7* and *qPT10–5*) were responsible for pericarp thickness located on chromosomes 7 and 10, respectively, explaining a total of 21.76% of phenotypic variance in 2016. Two QTLs were detected based on the average pericarp thickness of 3 years, which explained 3.22 and 35.38% of phenotypic variance. A stable *qPT10–5,* which was located in a 901.2-kb (chr10: 145,172,996-145,532,401) region, was identified in all 3 years and averaged across 3 years and could explain 7.78 to 35.38% of phenotypic variations among different years. This finding indicated that *qPT10–5* was the stable major locus interval controlling sweet corn pericarp thickness (Fig. 1; Table 2). According to the reference genome annotation information, forty-two genes within the region of *qpt10–5* were found.

**Table 2:** QTL detected for pericarp thickness in the BC_4_F_3_ population

Trait	QTL name	Chr^a^	CI (bp)^b^	QTL region (cM)	LOD^c^	ADD^d^	R^2^(%)^e^
2014FS	*qPT1–1*	1	289,632,655–291,951,018	292.01–296.98	2.76	12.37	6.76
	*qPT1–2*	1	294,334,812–295,560,828	273.97–274.01	2.27	3.02	3.36
	*qPT10–1*	10	73,141,877–74,489,414	0–5.39	2.72	13.02	6.72
	*qPT10–2*	10	51,051,991–53,058,997	34.48–35.56	2.22	12.97	6.64
	*qPT10–3*	10	49,740,097–50,781,644	89.26–89.32	2.16	12.96	6.69
	*qPT10–4*	10	31,389,610–33,625,026	105.62–105.73	2.1	12.95	6.65
	*qPT10–5*	10	144,631,242–145,532,401	233.54–233.57	3.27	14.00	7.78
2015FS	*qPT5*	5	14,148,610–18,559,963	39.86–42.01	2.26	14.66	7.62
	*qPT6*	6	117,425,264	204.24	2.92	16.69	9.88
	*qPT10–5*	10	144,631,242–145,532,401	233.54–233.57	4.12	15.74	8.82
2016FS	*qPT7*	7	67,564,733–69,008,076	231.99–232.04	2.21	14.62	7.92
	*qPT10–5*	10	144,631,242–145,532,401	233.54–233.57	7.13	22.61	13.84
Average FS	*qPT1–3*	1	295,869,898	286.16	2.29	2.42	3.22
	*qPT10–5*	10	144,631,242–145,532,401	233.54–233.57	4.34	30.40	35.38

^a^Chr chromosome

^b^The physical position corresponding to the 95% confidence interval for the detected QTL

^c^LOD the logarithm of odds score

^d^Positive and negative values indicated additive effects by the alleles of M03 and M08, respectively

^e^R^2^ the phenotypic variance explained by an individual QTL

### Transcriptome profiling

**Fig. 2:**
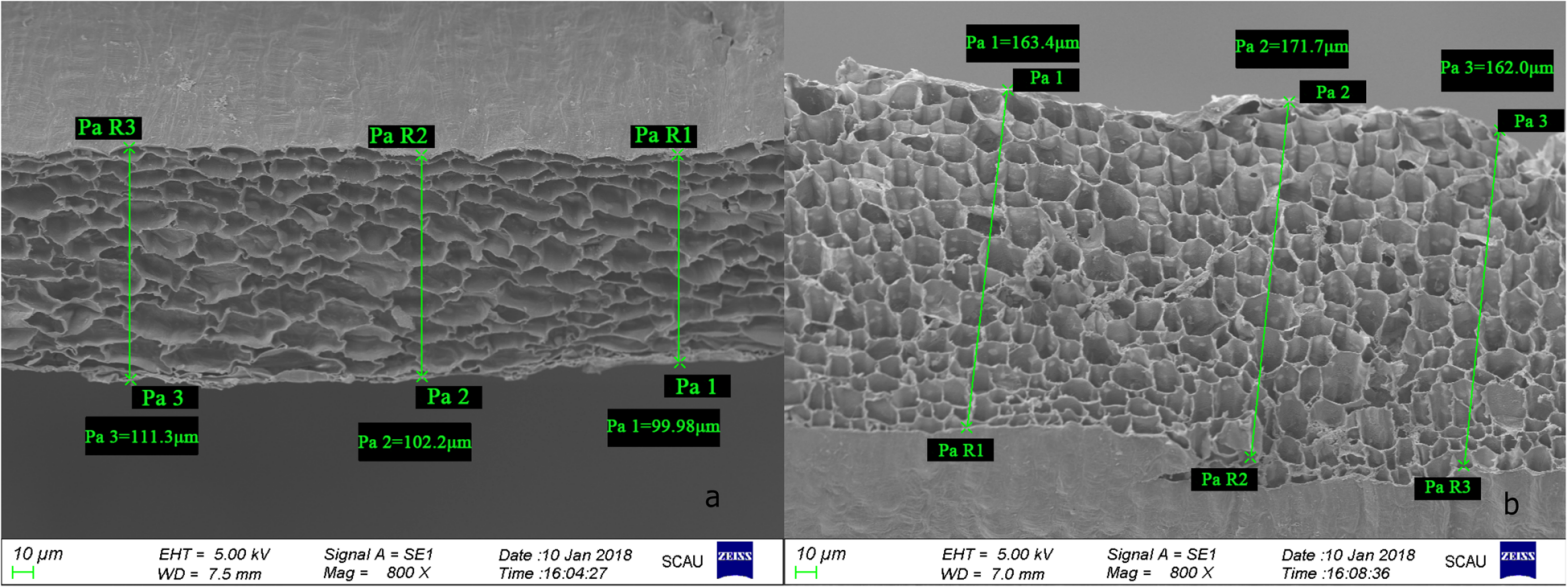
Scanning electron microscopy of pericarp thickness of M03 and M08. a, b Pericarp thickness of M03 and M08 at 19 DAP, respectively

To identify the differentially expressed genes (DEGs) in *qPT10–5*, the pericarp 19 days after pollination (DAP) for two parents was used for transcriptome sequencing. Scanning electron microscopy (SEM) analysis of the pericarps of the M03 and M08 lines (each sample with five replications) at the 19th DAP was also performed, and the average pericarp thicknesses of M03 and M08 were 111.27 ± 9.19 μm and 176.90 ± 13.86 μm, respectively (Fig. 2). Approximately 43.98 Gb of total nucleotide data were obtained from the M03 and M08 lines by RNA sequencing. Three independent biological replicates were used in this experiment. We obtained 49,153,314–54,655,798 reads for the inbred line M03, and 67.77 to 69.94% were mapped to the B73 reference genome (version 3). For the inbred line M08, we obtained 51,359,308-54,932,698 reads, and 70.06 to 70.46% were mapped to the B73 reference genome (Additional file 4: Table S3). A significantly high correlation (Additional file 5: Table S4) was observed between the two biological replications, revealing steady expression profiling between the replicated samples. In this study, a total of 4381 DEGs were identified between the M03 and M08 lines (|fold change| ≥ 2 and FDR < 0.01). Among these genes, 2318 were upregulated and 2063 were downregulated in the M03 line.

**Fig. 3:**
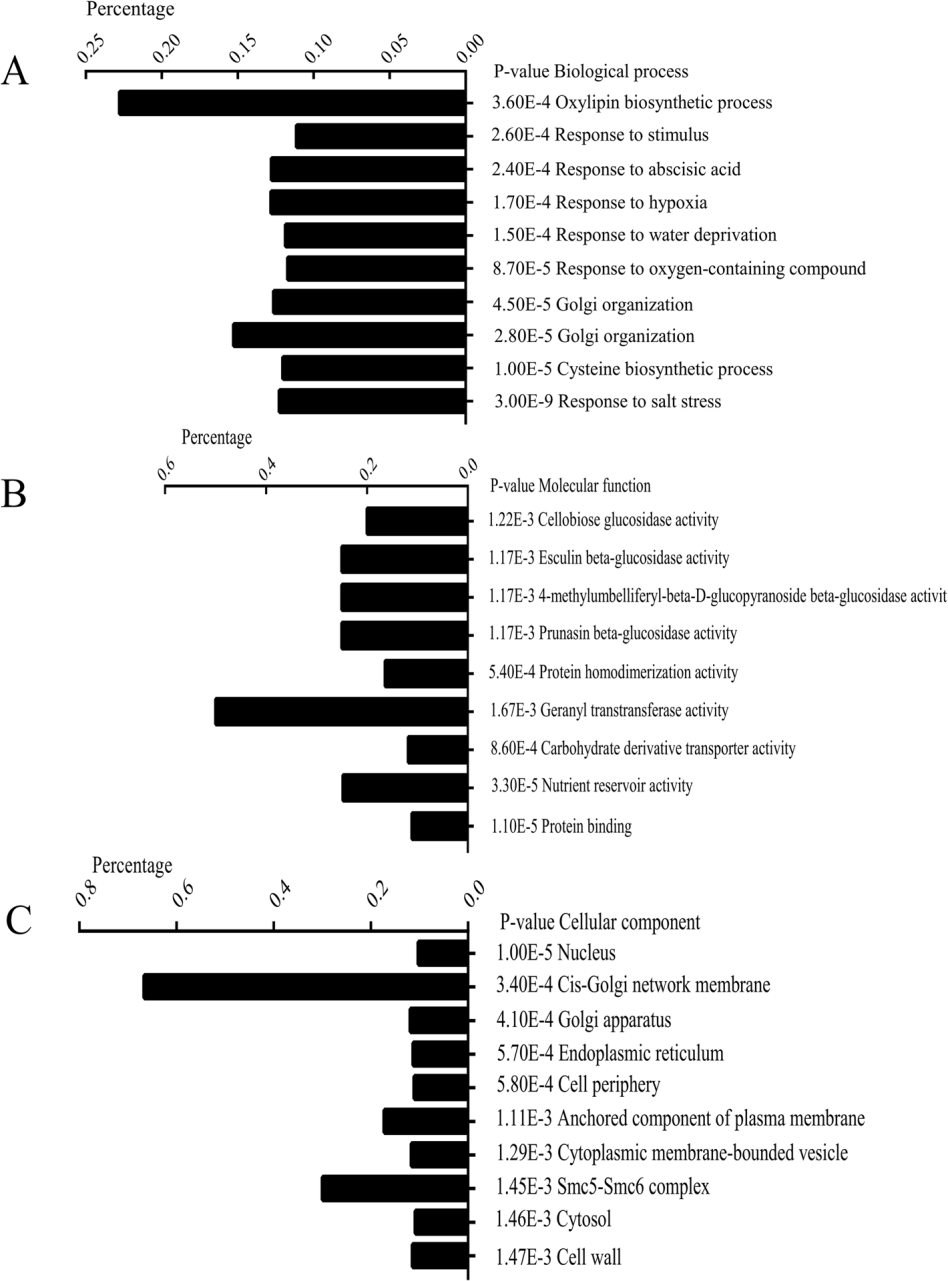
Gene ontology (GO) enrichment of the differentially expressed genes (DEGs) (*p* < 0.005). GO enrichment was performed using agriGO. **a** Biological process, **b** molecular function, and **c** cellular component. The percentage is the ratio of enriched DEGs to all genes in a given GO term using maize reference genes as background. The *P*-value denotes the enriched levels in a GO term, which were calculated using Fisher’s exact test

Gene Ontology-based enrichment analysis was carried out using a threshold value (FDR < 0.01) to evaluate the major biological functions of the DEGs (Fig. 3). These genes were further classified into three main categories, including biological processes (BP), cellular components (CC) and molecular functions (MF). The BP category contained half of the GO annotations (18,418; 50.96%) followed by the CC category (12,965; 35.87%) and the MF category (4757; 13.17%). Under the biological processes category, cysteine biosynthetic process, response to salt stress, response to cadmium ion, responses to stimulus, Golgi organization, response to oxygen-containing compound, response to water deprivation, response to hypoxia, response to abscisic acid, and oxylipin biosynthetic process were prominently represented. Within the cellular components category, nucleus, cis-Golgi network membrane, Golgi apparatus, endoplasmic reticulum, cell periphery, anchored component of plasma membrane, cytoplasmic membrane-bounded vesicle, Smc5-Smc6 complex, cytosol, and cell wall represented most of the subcategories. For the molecular functions category, many genes were classified into the protein binding, nutrient reservoir activity, carbohydrate derivative transporter activity, geranyltrans transferase activity, protein homodimerization activity, prunasin beta-glucosidase activity, 4-methylumbelliferyl-beta-D-glucopyranoside beta-glucosidase activity, esculin beta-glucosidase activity, and cellobiose glucosidase activity subcategories.

**Fig. 4:**
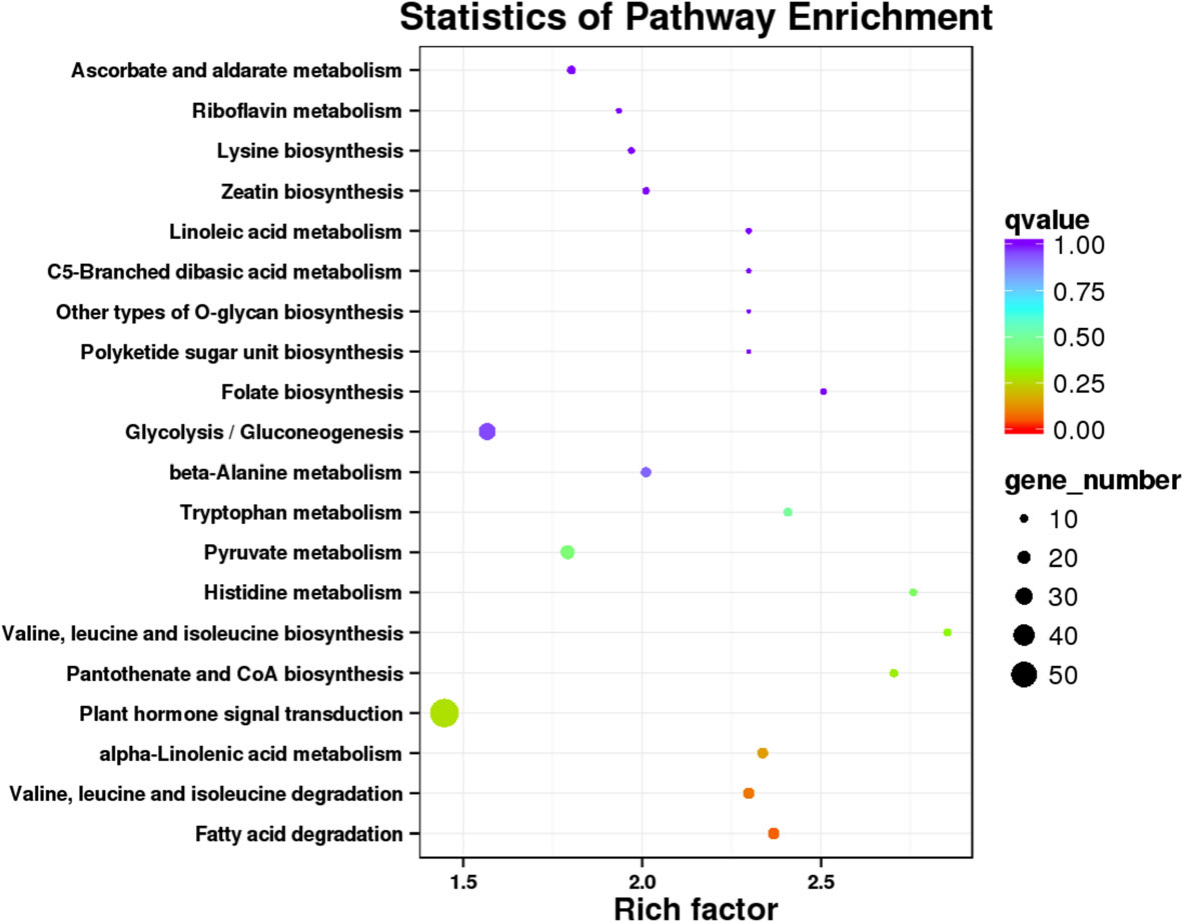
Enriched KEGG pathways of target genes for differentially expressed genes. The most enriched pathway is the plant hormone signal transduction pathway

KEGG enrichment analysis showed (FDR < 0.01) that these genes were mainly located in plant hormone signal transduction, glycolysis/gluconeogenesis, pyruvate metabolism, valine, leucine and isoleucine degradation and fatty acid degradation pathways (Fig. 4).

### Candidate gene prediction in *qpt10–5* based on transcriptome analysis

**Fig. 5:**
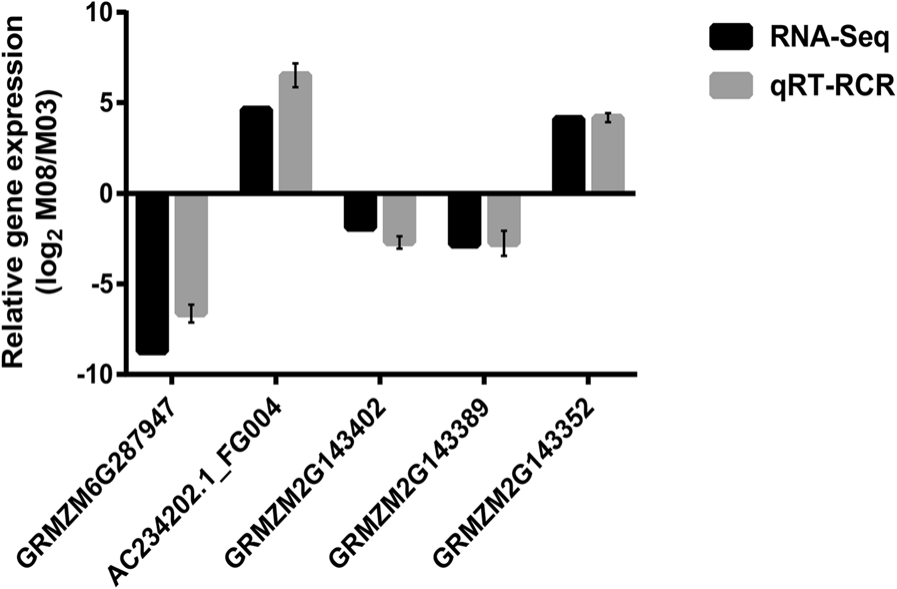
Verification of relative expression levels of DEGs by qRT-PCR. Error bars indicate standard deviation from 3 biological and technical replicates of qRT-PCR. Expression patterns of 10 DEGs by qRT-PCR (black bar) and RNA-Seq (grey bar)

**Fig. 6:**
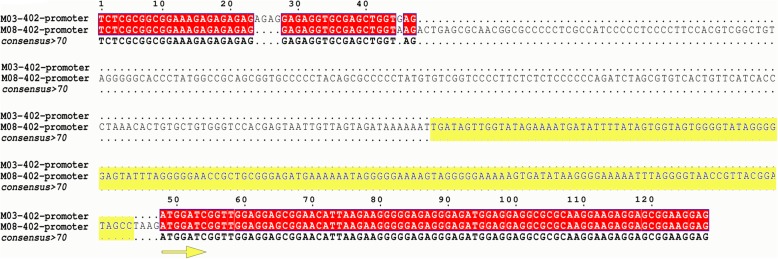
Promoter sequence difference in the *GRMZM143402* gene between M03 and M08. (*GRMZM2G143402* in M08 has a 362-bp insertion in the promoter, which contains a PIF-Harbinger transposon as indicated by the yellow sequence)

Forty-two candidate genes in the *qpt10–5* region were compared with the DEGs identified by transcriptome sequencing. Among the 42 genes, 18 were found in the transcriptome sequencing (Additional file 6: Table S5). Only five genes, *GRMZM6G287947*, *AC234202.1_FG004*, *GRMZM2G143402*, *GRMZM2G143352* and *GRMZM2G143389*, were differentially expressed between the M03 and M08 lines (Table 3). Gene annotation indicated that *GRMZM2G143352*, *GRMZM2G143402*, and *GRMZM2G143389* may be candidate genes that control pericarp thickness. Of these genes, *GRMZM2G143352* and *AC234202.1_FG004* were upregulated in the M08 line, whereas *GRMZM2G143389*, *GRMZM6G287947*, and *GRMZM2G143402* were downregulated in the M08 line. To confirm the results obtained from transcriptome sequencing, these genes were selected for qRT-PCR validation. As expected, the expression patterns of the 5 selected genes obtained from qRT-PCR were similar to those obtained by transcriptome sequencing (Fig. 5). Furthermore, the full-length versions of those three genes in the M03 and M08 lines were sequenced. *GRMZM2G143352* in the M08 line was missing a CGCG and ACCTCG sequence in front of the initiation codon and coding sequence compared with the sequence from the M03 line. Although the coding sequence was the same, *GRMZM2G143402* in the M08 line had a 362-bp sequence insertion in the promoter, which contained a PIF-Harbinger transposon (Fig. 6). *GRMZM2G143389* had a copy of CCGCTCA in the promoter of the M08 line compared with M03 (Additional file 7: Fig. S2). Variation in these three genes may lead to differences in pericarp thickness between the two parents. These results may facilitate the fine mapping of the *qPT10–5* locus, and further experiments are needed to identify functional genes and identify causes of the differences in pericarp thickness.

**Table 3:** Different expressed genes within the interval of *qPT10–5* in sampled pericarp for M03 and M08

Gene ID	M03^a^	M08^a^	FDR	Log_2_FC^b^	Regulated^c^	Gene Annoation
GRMZM2G143352	0.29	4.4	8.33E-05	4.10	up	Auxin-responsive protein IAA33
GRMZM2G143389	17.21	2.53	4.88E-07	−2.83	DOWN	Protein FATTY ACID EXPORT 2 chloroplastic
GRMZM2G143402	132.88	36.08	2.19E-09	−1.89	DOWN	ZIM-transcription factor 34
GRMZM6G287947	202.77	0.68	8.76E-14	−8.73	DOWN	None
AC234202.1_FG004	0	2.51	1.64E-12	Inf	up	Hypothetical protein ZEAMMB73_137218

^a^The average expression level is FPKM value

^b^Fc FPKM change between M03 and M08

^c^*NS* Non-significant, *UP* Upregulated expression, *DOWN* Downregulated expression

## Discussion

### Characteristics of the SLAF-seq method

Compared to other sequencing techniques combined with restriction enzyme digestion (such as genotyping-by-sequencing and restriction-site-associated DNA sequencing), SLAF-seq was measured by sequencing the specific length amplified fragment, which has better repeatability. Exploring numerous stable and reliable molecular markers is the key step for high-density genetic map construction. Once the genome is digested by restriction endonuclease(s), choosing fragments correctly for sequencing would be a better representation of the genome. In this study, the total number of SLAF markers was more than 520,000, and the number of polymorphic markers was 31,582. This result is difficult to achieve with traditional molecular markers. Furthermore, all the markers covered the whole genome, which ensured the accuracy of the final mapping. The labels with poor representativeness and inadequate completeness and the bias separation labels could be filtered out. All of these beneficial properties simplified the genome sequencing, which proves the superiority of this technique in genetic mapping. On the other hand, due to the large number of markers, complex populations and other factors, the sequencing results also increased the difficulty and challenge of the mapping analysis. Although the construction of the linkage map and its application in common corn have been widely applied [22, 23], few studies have been published about genetic mapping of the whole genome in sweet corn. In this study, we provide a reference for follow-up genetic research on sweet corn.

### Pericarp thickness QTL mapping results compared with other studies

With the rapid development of molecular biotechnology and bioinformatics, plant gene mapping has made considerable progress. Previous studies have shown that a total of 190 BC_1_F_2_ families crossed by two cultivars with different pericarp thicknesses were used as the mapping population. Eight QTLs were found to be linked to pericarp thickness and mapped to chromosomes 1, 2, 3, 5, 6 and 8 [12]. In addition, 33 QTLs were detected by 264 F_2:3_ families crossed by the Korean waxy maize inbred lines BH20 and BH30, and these loci were located on chromosomes 1, 2, 3, 4, 8, 9, and 10 [11]. In this study, based on the previous research conclusions, pericarp thickness of the upper abgerminal part was regarded as a representative to determine the sweet corn pericarp thickness. Finally, a major QTL locus was found on chromosome 10 and could explain 7.78 to 13.84% of phenotypic variations among different years. Phenotypic variance explained by a QTL was estimated based on the same data that were used to detect the QTL, which could cause the R^2^ values to be biased upwards [24]. Among the chromosomes, 10 chromosomes had the most frequently linked markers. This result is different from that of Yu et al. but is similar to that of Choe and Rocheford [11, 12]. It was found that the loci mapped on the same chromosome were generally adjacent to their predecessors. Therefore, the chromosome may have the same major gene loci as previous generations.

### Candidate gene analysis

*GRMZM2G143352* is an AUX/IAA transcription factor, *GRMZM2G143402* is a ZIM transcription factor, and *GRMZM2G143389* is the chloroplastic FATTY ACID EXPORT 2 protein. Both *GRMZM2G143352* and *GRMZM2G143402* were related to plant hormone signal transduction, and *GRMZM2G143389* was related to fatty acid degradation pathways, which is consistent with the results of the KEGG enrichment analysis (Fig. 4). Furthermore, the full-length gene sequences of these three genes are different between the M03 and M08 lines. Variation in these three genes may lead to differences in pericarp thickness between the two parents.

*GRMZM2G143352* is an AUX/IAA transcription factor that mediates many aspects of plant responses to auxin [25]. The functions of most Aux/IAAs have been defined mainly by gain-of-function mutant alleles in *Arabidopsis thaliana* [26]. Two different classes of transcription factors are involved in the regulation of auxin signalling, AUX/IAAs and AUXIN RESPONSE FACTORS (ARFs). ARFs bind directly to auxin response elements in the promoter region of auxin-responsive genes via their conserved DNA-binding domain (DBD). When auxin is low, AUX/IAA proteins bind to ARFs, preventing the expression of auxin-responsive genes. A high level of auxin promotes ubiquitination and degradation of AUX/IAAs though SCF^TIR1/AFB^ and the 26 s proteasome, leading to the activation of auxin-response genes by ARFs [27, 28]. These response genes may be related to pericarp development.

We know that auxin treatment can promote the expression of AUX/IAA genes [29, 30]. We found that the expression level of *GRMZM2G143352* in the M08 line was higher than that in the M03 line, indicating that the auxin level of the M08 line could be higher than that in the M03 line. Auxin is an important cell cycle regulator [31]. The level of auxin affects cell growth, lowers concentrations of indole-3-acetic acid (IAA) and can promote the elongation of tobacco BY-2 cells but has no effect on cell division. However, cell division was accelerated, but cell elongation was inhibited at relatively high IAA concentrations [32, 33]. These findings are consistent with our results (Fig. 3).

*GRMZM2G143402* is a ZIM transcription factor that is a repressor of JA (jasmonate) in maize [34]. The JA signal regulates plant growth, development and defence responses. JAZ (jasmonate ZIM domain) transcription repressors bind directly to JA-responsive genes. Without the JA ((3R,7S)-jasmonoyl-L-isoleucine) signal, JAZ regulates the JA-mediated response by inhibiting the transcriptional activity of JA signalling-responsive transcription factors [35, 36]. When the JA signal appeared, the JA receptor specifically combined with JAZ, resulting in JAZ ubiquitination and degradation by proteasomes, relieving JAZ’s inhibition of JA transcriptional regulation and causing physiological changes [37]. Through regulating the development of the stomata, JA can improve the ability of plants to cope with various external stresses [38]. Some components of the JA signalling pathway can be independently involved in plant stomatal development regulation [39]. The size of the stoma and the open state may be one of the reasons for the difference in pericarp thickness.

According to the gene annotation, *GRMZM2G143389* is the fatty acid export 2 (*FAX2*) chloroplastic protein. Fatty acid synthesis in plants occurs in plastids; therefore, it needs to export for acyl editing and lipid assembly in the cytoplasm and endoplasmic reticulum. However, plastid fatty acids’ transport mechanism has not been elucidated. The function of *fatty acid output 1* (*fax1*) is essential for biomass and male reproductive capacity [40], while *FAX2* function is unknown. Hence, it would be highly interesting if we could prove the association of this gene with sweet corn pericarp thickness. Therefore, additional evidence is required to demonstrate the potential role of these genes in the pericarp thickness of sweet corn.

## Conclusions

In our study, we created a BC_4_F_3_ population and constructed a high-density genetic linkage map that had an average genetic distance of 0.62 cM between adjacent markers by the SLAF approach. Based on this high-density genome map, a total of 14 QTLs for pericarp thickness were detected, and one stable QTL (*qPT10–5)* was detected across multiple years, which explained 7.78–35.38% of the phenotypic variation located on chromosome 10. Moreover, five genes of the target region of *qPT10–5* were differentially expressed between the two parents, as revealed by transcriptome analysis. According to the gene annotation information, three genes might be considered candidates for pericarp thickness. This study identified a major QTL and candidate genes that could accelerate breeding for thin pericarp thickness varieties of sweet corn, and these results established the basis for map-based cloning and further functional research.

## Methods

### Plant material

Two sweet corn inbred lines, M03 and M08, were selected as parents to develop a BC_4_F_3_ mapping population. The two lines show different pericarp thicknesses; as the recurrent parent, M03 is a thinner pericarp thickness; as the donor parent, M08 is a thicker pericarp thickness line. All plant materials used in this study were generously provided by Prof. Xiaoqin Li (College of Agriculture, South China Agricultural University, Guangzhou, China). The mapping population consisted of 148 lines, and evaluation of background recovery rates showed only 8 lines whose genetic background recovery rates were less than 90%; the recovery rates of the other lines were higher than 90%, the highest rate was 99.99%, and the average background recovery rate was 95.91%. These results showed that the genetic background of the BC_4_F_3_ population was largely the same as that of the recipient parents.

Two parents and 148 lines were grown in the Zengcheng Experimental Base of South China Agricultural University (at approximately 113° east longitude and approximately 23° north latitude) in autumn of 2014, 2015, and 2016. Two parents planted in 2017 were used for transcriptome sequencing. A randomized complete-block design was used. Each line or parent was grown in 10 plants with 2 replicates. The length of the rows was 3 m, and the row spacing was 70 cm. The plant spacing was 25 cm, and the density was 57,000 hm^2^. Crop management and disease and insect pest control were performed as locally recommended.

Three self-pollinated ears of the two parents were sampled and placed immediately on ice 19 days after pollination in the field in 2017. Ten kernels were collected from the middle of each ear. The upper abgerminal part of the pericarp was stripped on ice and stored in liquid nitrogen for analysis by scanning electron microscopy.

### Phenotypic data collection

#### Frozen section method

Ten whole kernels from each ear were placed on ice. Each kernel was cut off approximately 3 mm at the top with bald tweezers and then transferred into liquid nitrogen for 3 s. Frozen samples were used for the preparation of tissue slices. We took cross-sections from the dorsal embryo with a thickness of approximately 100 μm. The process was completed before thawing to ensure that the pericarp did not fall off. The slices were then quickly placed on the slide. After thawing, KI-I_2_ reagent was added for 3 s, and the dye was then washed off with water after covering the coverslip. The pericarp thickness of each kernel was measured by micrometer. The average value of pericarp thickness from three ears of each genotype was regarded as the observed value of the pericarp thickness and used for subsequent analysis [2, 41].

### Phenotypic data analysis

The phenotypic data were analyzed using SPSS version 19 (SPSS Inc., Chicago, IL, USA). These variance components of the genotype and environment in each year were estimated by using the linear mixed model: y_il_ = *μ* + *r*_*l*_ + *f*_*i*_ + *ε*_*il*_, and these variance components of the genotype and environment in 3 years were estimated by using the following mixed model: y_ijl_ = *μ* + *e*_*j*_ + *r*_*l*(*j*)_ + *f*_*i*_ + (*fe*)_*ij*_ + *ε*_*ijl*_,where *μ* is the grand mean of pericarp thickness, *f*_*i*_ is the genetic effect of the *i*th line, *r*_*l*_ is the effect of replications, *ε*_*il*_ is the residual error, *e*_*j*_ is the environmental effect of the *j*th environment, (*fe*)_*ij*_ is the interaction effect between genetic and environmental effects, *r*_*l*(*j*)_ is effect of replications within environments, and *ε*_*ijl*_ is the residual error [42]. The PROC MIXED procedure in SAS software (Release 9.4; SAS Institute, Cary, NC) was used to get the variance components of all pericarp thickness traits.

The broad sense heritability (*h*^2^) in each year was estimated using the following formula: ${h}^2={\sigma}_g^2/\left({\sigma}_g^2+{\sigma}_z^2/r\right)$, and the ${h}_b^2$ in cross-years was estimated using the following formula: ${h}_b^2={\sigma}_g^2/\left({\sigma}_g^2+{\sigma}_{ge}^2/e+{\sigma}_z^2/ re\right),$ where *σ*_*g*_^2^ is the genetic variance, *σ*_*z*_^2^ is the residual error, and r is the number of replications, *σ*_ge_^2^ is the interaction of genotype with environment, *e* and *r* represent the number of environments and replications in each environment [42].

### Library construction and high-throughput sequencing

Genomic DNA was extracted from the two parents and the BC_4_F_3_ population using a cetyl trimethylammonium bromide (CTAB) protocol [43]. Illumina HiSeq™ 2500 sequencing was used for DNA sequencing. The Hpy166II endonuclease was chosen to digest the genomic DNA after profiling the restriction endonuclease sites on the reference genome. Details of the SLAF-seq strategy and library construction were described previously [13].

### Grouping and genotyping of sequence data

Specific length amplified fragment (SLAF) markers were identified and genotyped following procedures described by Sun et al. [13] and Zhang et al. [44]. The remaining reads were mapped to the reference genome using BWA software after filtering out the low-quality reads [45]. Sequences that had greater than 95% similarity were defined as the same SLAFs. All polymorphic SLAF markers that were consistent with parents and offspring were genotyped.

All SLAF markers were filtered four times, and the quality was assessed as described by Sun et al. [13]. In brief, markers with fewer than 3 SNPs and an average sequencing depth higher than 3 were treated as high-quality SLAF markers. These markers were used to construct high-density genetic maps.

### Linkage map construction

The imputation of missing genotypes was performed using the K-nearest neighbour algorithm based on the two parents and the BC_4_F_3_ population [46]. A linkage map was constructed according to the procedure described by Zhang et al. [44]. In detail, the SLAF markers in the linkage groups were ordered using the High Map Strategy [47] and SMOOTH strategy [48]. Multipoint method of maximum likelihood was used to add these skewed markers into the linkage map [49]. The genetic distance between the adjacent markers was estimated by kosambi mapping function [50].

### QTL analysis

QTL mapping was carried out by the composite interval mapping method in QTLNetwork v2.1 software for a pericarp thickness of three years and an average of three years [51]. 1000 permutations with the mapping step of 1.0 cM was applied to calculate the LOD threshold. By default, a 10 cM window with background marker set to 5, and a genome-wide significance level of *P* < 0.05 [52]. QTLs with the LOD score > 2.0 were considered as suggestive QTLs [14]. Moreover, the mode of QTL action was determined according to the criteria proposed by Stuber et al. [53]. The QTL is named by q and abbreviated by traits. In addition, the chromosome number of the QTL and multiple QTLs on the same chromosome are designated by 1, 2, 3, and so on. The QTL names in this paper are expressed in italics; for example, *qpt1–3* indicates the third QTL on chromosome 1 detected in the controlled pericarp thickness population.

### Transcriptome analysis

Total RNA was isolated from the pericarps (19th DAP) using the Plant Total RNA Purification Kit (TR02–150, GeneMarkbio). The extracted RNA was run in a 1% agarose gel to assess the integrity of the RNA. Briefly, poly-A RNA-containing mRNA was enriched using poly-Toligo-attached magnetic beads and fragmented. Second-strand cDNA was synthesized using random hexamer primers and then purified, end-repaired, poly-A tailed, and adaptor-ligated. The cDNA libraries were sequenced with a read length of 100 bp (paired-end) using the Illumina HiSeq 2000 System at Biomarker Technologies (Beijing, China). The experiment was performed with three biological replicates.

### Quantitative reverse-transcriptase PCR

Quantitative reverse-transcriptase PCR (qRT-PCR) was carried out to validate the RNA-seq results. The total RNA from each sample was extracted and reverse transcribed into single-stranded cDNA using a FastQuant RT Kit (Takara) including gDNase according to the manufacturer’s protocol. Gene expression was determined by qRT-PCR analysis using the CFX96 Real-Time System (Bio-Rad). All reactions were performed in 20-μL volumes consisting of 1 μl cDNA, 0.3 μM of each gene-specific primer and the SsoFast EvaGreen Supermix Kit (Bio-Rad). qRT-PCR was conducted with the following protocol: 94 °C for 1 min followed by 40 cycles of 95 °C for 10 s, 55 °C for 10 s, and 72 °C for 15 s. The relative transcriptional levels were calculated using the 2^−△△CT^ method [54] with actin as a housekeeping gene. The specific primers were designed using NCBI primer BLAST (http://www.ncbi.nlm.nih.gov/tools/primer-blast/). The primer sequences for each gene are listed in Additional file 8 (Table S6).

## Supplementary information

**Additional file 1: Table S1.** Correlation coefficients of pericarp thickness measured among the three years.

**Additional file 2: Fig. S1.** Genetic map of the 148 CSSLs. The black stripe shows the distribution of markers on the 10 chromosomes.

**Additional file 3: Table S2.** Basic information of the genetic map.

**Additional file 4: Table S3.** RNA-seq reads of sweet corn pericarp mapped to the maize B73 RefGen_V3 genome.

**Additional file 5: Table S4.** Pearson correlations for the RNA-sequencing results among different samples.

**Additional file 6: Table S5.** Expressed genes within the interval of *qPT10–5* in sampled pericarp for M03 and M08.

**Additional file 7: Fig. S2.** Promoter sequence difference in the *GRMZM143389* gene between M03 and M08. (The green box is a copy of CCGCTCA, and the yellow box has an inserted CTCGAGCAG sequence).

**Additional file 8: Table S6.** Primers for quantitative real-time RT-PCR (qRT-PCR) validation.

## Data Availability

All SLAF-seq raw data from this study has been submitted to the NCBI Sequence Read Archive (SRA) database under BioProject accession number PRJNA574257 (https://www.ncbi.nlm.nih.gov/bioproject/PRJNA574257). The raw RNA-sequencing data were deposited in NCBI SRA database under BioProject accession number PRJNA605850 (https://www.ncbi.nlm.nih.gov/bioproject/?term=PRJNA605850). The maize reference genome B73 RefGen_V3 sequence and annotation files were downloaded from ftp://ftp.ensemblgenomes.org/pub/plants/release-24/fasta/zea_mays/dna/ and ftp://ftp.ensemblgenomes.org/pub/plants/release-24/gtf/zea_mays/.

## Abbreviations

ARFs: Auxin response factors
CIM: Composite interval mapping method
DAP: Days after pollination
DEGs: Different expression genes
FAX1: Fatty acid export 1
FAX2: Fatty acid export 2
FS: Frozen section
IAA: Indole-3-acetic acid
JAZ: Jasmonate ZIM domain
LOD: Base 10 log likelihood ratio
QTL: Quantitative trait loci
RFLP: Restriction fragment length polymorphism
SEM: Scanning electron microscope
SLAF: Specific length amplified fragment
SNP: Single nucleotide polymorphism
SSR: Simple sequence repeats
