# Supplementary material for: QTL mapping and transcriptome analysis identify candidate genes regulating pericarp thickness in sweet corn
Source: BMC Plant Biol. 2020 Mar 14;20:117. doi: 10.1186/s12870-020-2295-8 (PMC7071591; doi:10.1186/s12870-020-2295-8)
Supplement: Supplementary file 2 — Additional file 2: Fig. S1. Genetic map of the 148 CSSLs. The black stripe shows the distribution of markers on the 10 chromosomes. [file 12870_2020_2295_MOESM2_ESM.pptx]

## Slide 1
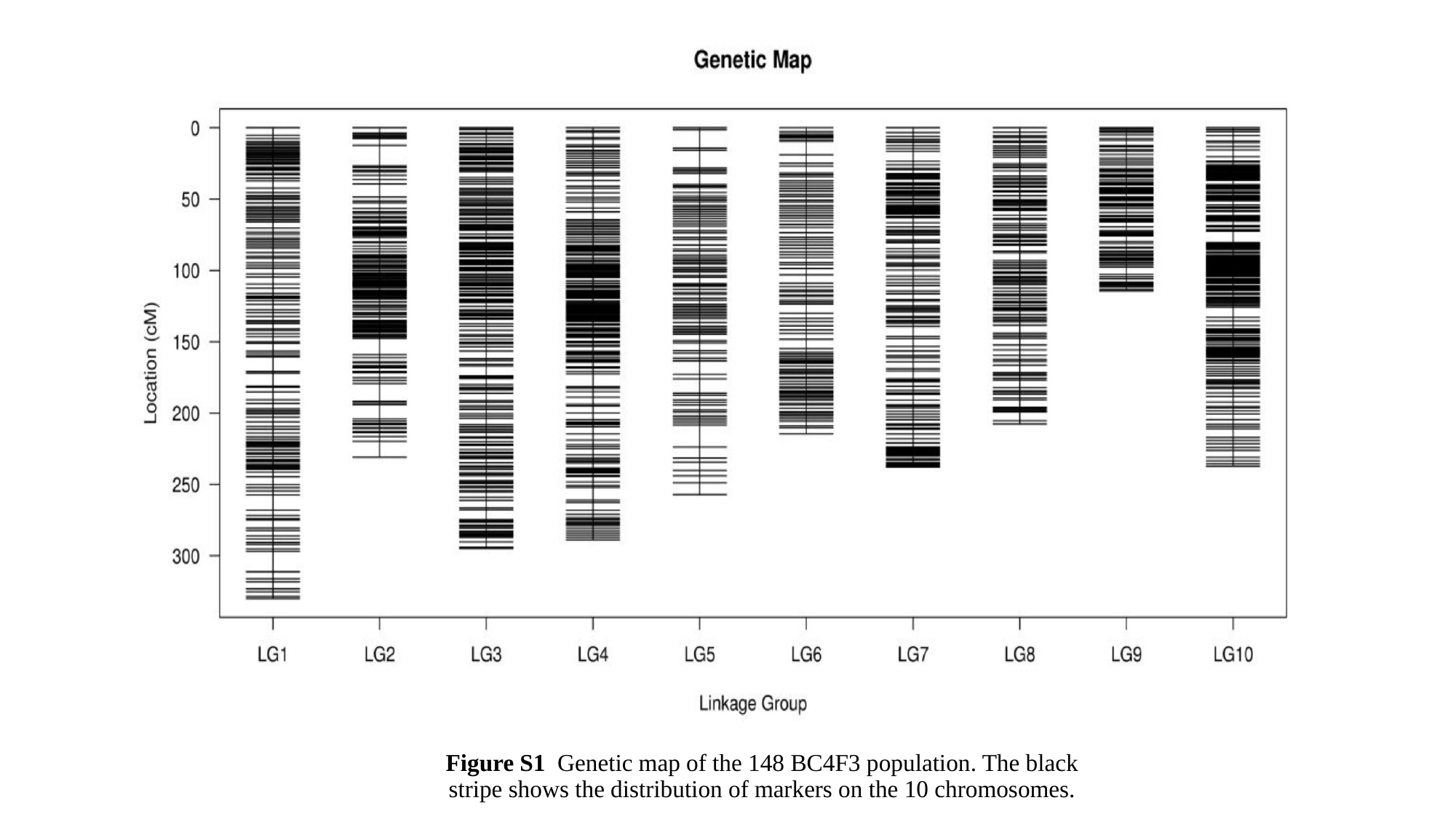

Figure S1 Genetic map of the 148 BC4F3 population. The black stripe shows the distribution of markers on the 10 chromosomes.
