# Supplementary material for: QTL mapping and transcriptome analysis identify candidate genes regulating pericarp thickness in sweet corn
Source: BMC Plant Biol. 2020 Mar 14;20:117. doi: 10.1186/s12870-020-2295-8 (PMC7071591; doi:10.1186/s12870-020-2295-8)
Supplement: Supplementary file 7 — Additional file 7: Fig. S2. Promoter sequence difference in the GRMZM143389 gene between M03 and M08. (The green box is a copy of CCGCTCA, and the yellow box has an inserted CTCGAGCAG sequence). [file 12870_2020_2295_MOESM7_ESM.pptx]

## Slide 1
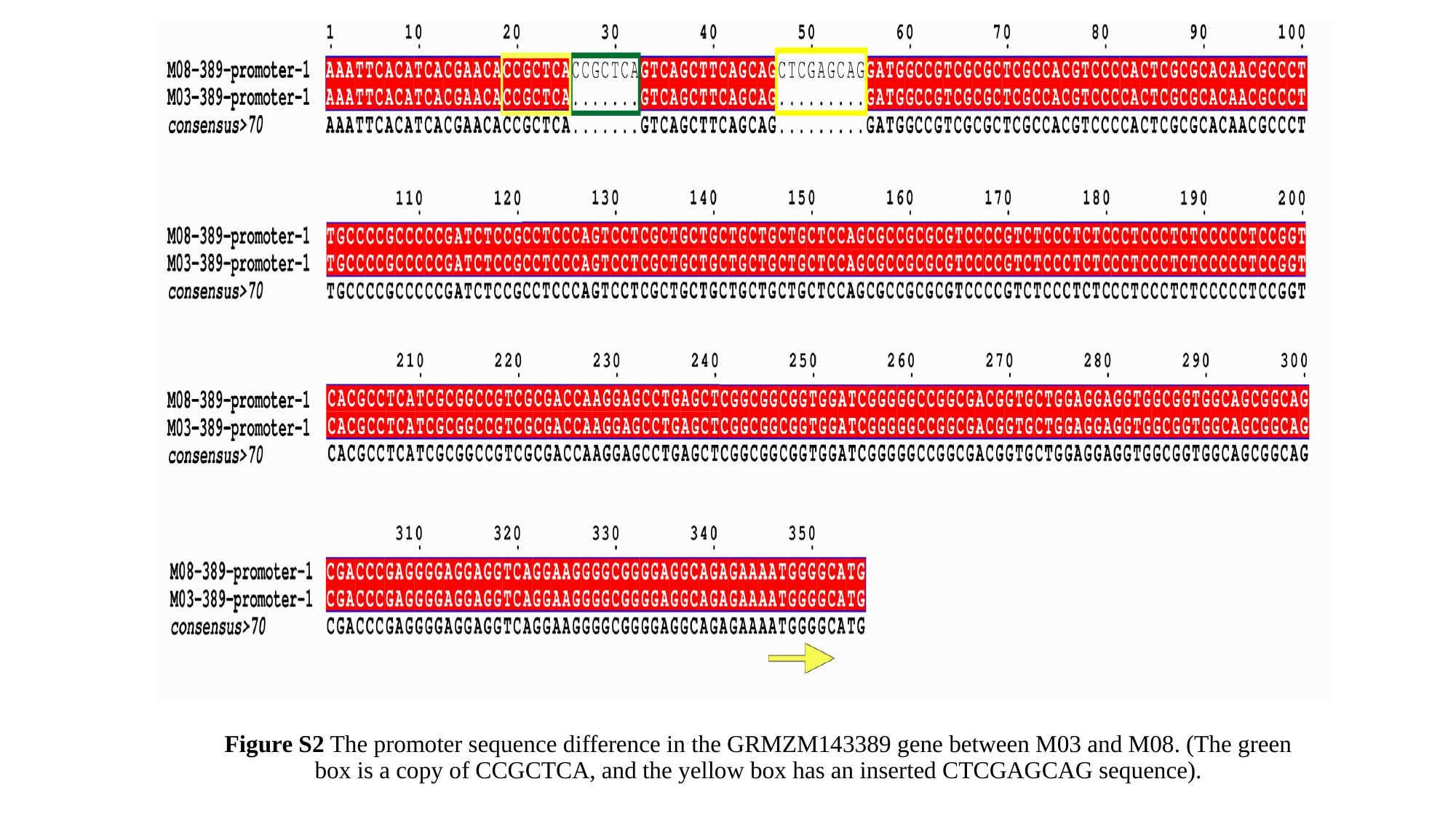

Figure S2 The promoter sequence difference in the GRMZM143389 gene between M03 and M08. (The green box is a copy of CCGCTCA, and the yellow box has an inserted CTCGAGCAG sequence).
